# Implantation of an Artificial Intelligence Denoising Algorithm Using SubtlePET™ with Various Radiotracers: 18F-FDG, 68Ga PSMA-11 and 18F-FDOPA, Impact on the Technologist Radiation Doses

**DOI:** 10.3390/jimaging11070234

**Published:** 2025-07-11

**Authors:** Jules Zhang-Yin, Octavian Dragusin, Paul Jonard, Christian Picard, Justine Grangeret, Christopher Bonnier, Philippe P. Leveque, Joel Aerts, Olivier Schaeffer

**Affiliations:** 1 Department of Nuclear Medicine, Centre National PET, Centre Hospitalier de Luxembourg, 4 Rue Ernest Barblé, L-1210 Luxembourg, Luxembourg; pauljonard@yahoo.fr (P.J.); csg.picard@yahoo.fr (C.P.); grangeret.justine@gmail.com (J.G.); bonnier.christopher@chl.lu (C.B.);; 2 Department of Medical Physics, Centre Hospitalier de Luxembourg, 4 Rue Ernest Barblé, L-1210 Luxembourg, Luxembourg; 3 Department of Radiopharmacy, Centre Hospitalier de Luxembourg, 4 Rue Ernest Barblé, L-1210 Luxembourg, Luxembourg; philippe.leveque@chem.lu (P.P.L.);

**Keywords:** AI-based denoising, radiotracer dose reduction, PET/CT imaging, technologist radiation exposure

## Abstract

This study assesses the clinical deployment of SubtlePET™, a commercial AI-based denoising algorithm, across three radiotracers—^18^F-FDG, ^68^Ga-PSMA-11, and ^18^F-FDOPA—with the goal of improving image quality while reducing injected activity, technologist radiation exposure, and scan time. A retrospective analysis on a digital PET/CT system showed that SubtlePET™ enabled dose reductions exceeding 33% and time savings of over 25%. AI-enhanced images were rated interpretable in 100% of cases versus 65% for standard low-dose reconstructions. Notably, 85% of AI-enhanced scans received the maximum Likert quality score (5/5), indicating excellent diagnostic confidence and noise suppression, compared to only 50% with conventional reconstruction. The quantitative image quality improved significantly across all tracers, with SNR and CNR gains of 50–70%. Radiotracer dose reductions were particularly substantial in low-BMI patients (up to 41% for FDG), and the technologist exposure decreased for high-exposure roles. The daily patient throughput increased by an average of 4.84 cases. These findings support the robust integration of SubtlePET™ into routine clinical PET practice, offering improved efficiency, safety, and image quality without compromising lesion detectability.

## 1. Introduction

Positron Emission Tomography (PET) is a pivotal imaging modality extensively used in oncology, neurology, and cardiology for its high sensitivity and ability to provide detailed functional information about various pathophysiological processes [1]. However, the increasing demand for PET scans has posed significant challenges in terms of resource management and patient throughput [2]. Additionally, the inherent limitations of PET, such as the need for high injected activity and long acquisition times to achieve optimal image quality, further strain clinical workflows and patient comfort [3].

Recent advancements in digital PET technology, particularly those using digital photon counting (DPC) and incorporating silicon photomultipliers (SiPMs), have enhanced the timing, energy, and spatial resolution of PET systems, contributing to faster scanning capabilities and potentially reduced injected activities [4]. Despite these technological improvements, there remains a compelling need to further optimize PET imaging to meet the growing demand without compromising the image quality [5].

Artificial intelligence (AI) and deep learning (DL) have emerged as transformative tools in medical imaging, offering innovative solutions to enhance the image quality through advanced denoising algorithms. AI-based denoising, particularly using deep convolutional neural networks (DCNNs), has shown promise in maintaining or even improving the image quality while allowing for reduced acquisition times and lower injected doses [6]. SubtlePET™, a commercially available AI-based denoising software, has been validated in several studies for its efficacy in improving the quality of low-dose PET images [7,8,9].

Previous studies have demonstrated the feasibility and benefits of AI-based denoising procedures, using SubtlePET™, in various clinical settings. Weyts et al. investigated the potential of AI to halve 18F-FDG PET acquisition times without degrading the image quality, finding high concordance rates and comparable image metrics between AI-denoised and standard images [10]. Bonardel et al. confirmed the clinical applicability of SubtlePET™ for reducing acquisition times and injected doses while maintaining diagnostic accuracy in FDG PET imaging [11]. Additionally, Margail et al. highlighted the utility of AI denoising for 68Ga-PSMA11-PET scans, commonly used in prostate cancer imaging, achieving a significant noise reduction and image enhancement [12]. Furthermore, the feasibility of optimizing the use of 68Ge/68Ga generators through AI-based denoising to preserve the image quality despite lower detection counts has been explored, underscoring the versatility and robustness of these AI tools [13].

This paper aims to further investigate and validate the use of AI-based denoising across a broader range of PET imaging applications, including three different tracers: 18F-FDG, 68Ga PSMA-11, and 18F-FDOPA, and the impact on the technologist’s radioprotection.

## 2. Materials and Methods

This retrospective monocenter study included patients aged 18 years or older who were referred to our center for PET imaging with three radiotracers: 18F-FDG, 68Ga-PSMA-11, and 18F-FDOPA.

PET/CT scans were conducted using a digital silicon photomultiplier (SiPM) PET/CT system (VEREOS, Philips Healthcare, Amsterdam, Netherlands) in accordance with EANM imaging guidelines. The PET/CT scan is part of the EARL accreditation program (for FDG and 68Ga). Patients fasted for at least 6 h prior to the injection of 18F-FDG, 6 h prior to the injection of 18F-FDOPA, while no fasting was required for 68Ga-PSMA-11. Radiotracer dosages followed standard protocols: 18F-FDG at 3 MBq/kg intravenously, 68Ga-PSMA-11 at 2 MBq/kg intravenously, and 18F-FDOPA at 2 MBq/kg intravenously.

Before each PET scan, a low-dose non-contrast-enhanced CT scan was acquired for attenuation correction and as an anatomical reference. CT scan parameters were 100–140 kV, with variable mAs according to a dose right index of 14 and an iterative reconstruction dose of 4.64 × 0.625 mm slice collimation, a pitch of 0.83, a rotation time of 0.5 s, a 3D modulation, a matrix of 512 × 512, and a voxel size of 0.97 × 0.97 × 3 mm^3^.

After implementing SubtlePET, we reduced the acquisition time by 25% and more than 33% of injected activity, compared to conventional PET acquisitions performed using the standard protocol. Based on 20 randomly selected PET scans of each radiotracer, the visual image quality was assessed using a 5-point Likert scale based on the noise level, contrast, and signal-to-noise ratio (1 = uninterpretable, 5 = excellent). Images were classified as interpretable (score 3–5) or uninterpretable (score 1–2). Lesion detectability and diagnostic accuracy were assessed and compared between images acquired with and without SubtlePET.

For the quantitative analysis, the liver SUV_SD_ was utilized as a measure of the noise level.

The signal-to-noise ratio (SNR) was defined using the following formula:

$$SNR=\frac{{SUV}_{mean}(Liver\;VOI)}{SD(Liver\;VOI)}$$

The contrast-to-noise ratio (CNR) was defined using the following formula:

$$CNR=\frac{{SUV}_{mean,Lesion}-{SUV}_{mean,Liver}}{{SD}_{Liver}}$$

The volume of interest (VOI) was obtained as follows: a circular VOI with a diameter of 10 ± 2 mm was manually drawn in the homogeneous region of the liver, away from the edges and lesions. The SUVmean of the lesion was obtained by precisely contouring the lesion’s edge.

We analyzed 20 cases from the FDG group, 20 cases from the PSMA group, and 18 cases from the FDOPA group.

For the staff radiation dose measurement, we compared two periods: one during the conventional PET acquisitions following the standard protocol (before and therefore without SubtlePET) and another during the PET acquisitions processed using the SubtlePET™ algorithm. No shielding, workflow, or protocol changes were made during the study period before and after the SubtlePET™ implementation. Staff behavior and exposure were monitored under identical conditions, and the injection workflow remained unchanged.

The staff radiation dose was measured during all PET/CT procedures performed within our center during these two periods. During the procedure, the staff were advised to limit patient contact as well as they could and to stay at a reasonable distance from the patient during their work. An electronic personal dosimeter (EPD model MK2, APVL) was provided to all the staff to measure the received radiation doses. The EPD was carried ventrally on top of the clothing at a thoracic height. All the data has been collected and analyzed.

Data were analyzed to compare visual and semi-quantitative parameters between acquisitions without and with SubtlePET. Differences in SUV metrics were assessed using paired *t*-tests and a Bland–Altman analysis. The technologist radiation doses were evaluated using Wilcoxon signed-rank tests. Statistical significance was set at *p* < 0.05.

## 3. Results

### 3.1. Patient Characteristics

A total of 3946 PET/CT procedures were performed using three different radiotracers: FDG, PSMA, and FDOPA. The majority of examinations (*n* = 3816) utilized the FDG, primarily for general oncologic indications, including lung, breast, lymphoma, colorectal, and ENT cancers. The mean age of patients in the FDG group was 61.2 ± 11.0 years. The PSMA PET/CT was performed in 112 patients, with a mean age of 71.5 ± 7.5 years, exclusively for prostate cancer assessment. Finally, the FDOPA PET/CT was conducted on 18 patients (mean age 58.0 ± 13.0 years) for neuro-oncological and neuroendocrine tumor evaluations. These figures reflect the predominant use of the FDG in routine oncologic imaging, while the PSMA and FDOPA PET/CT were reserved for more specialized clinical indications. The characteristics of the patients are shown in Table 1.

### 3.2. Visual Analysis of Image Quality

**Figure 1:**
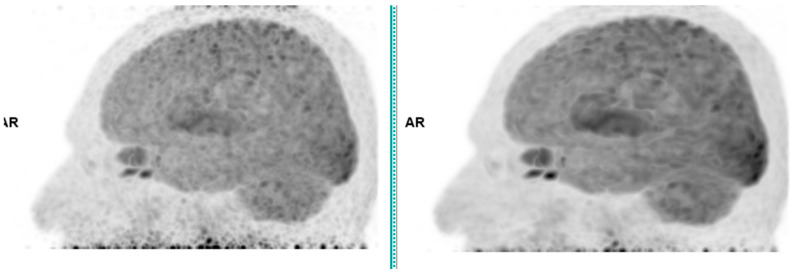
Brain 18F-FDG PET CT (**left**: without AI denoising; **right**: with AI denoising).

**Figure 2:**
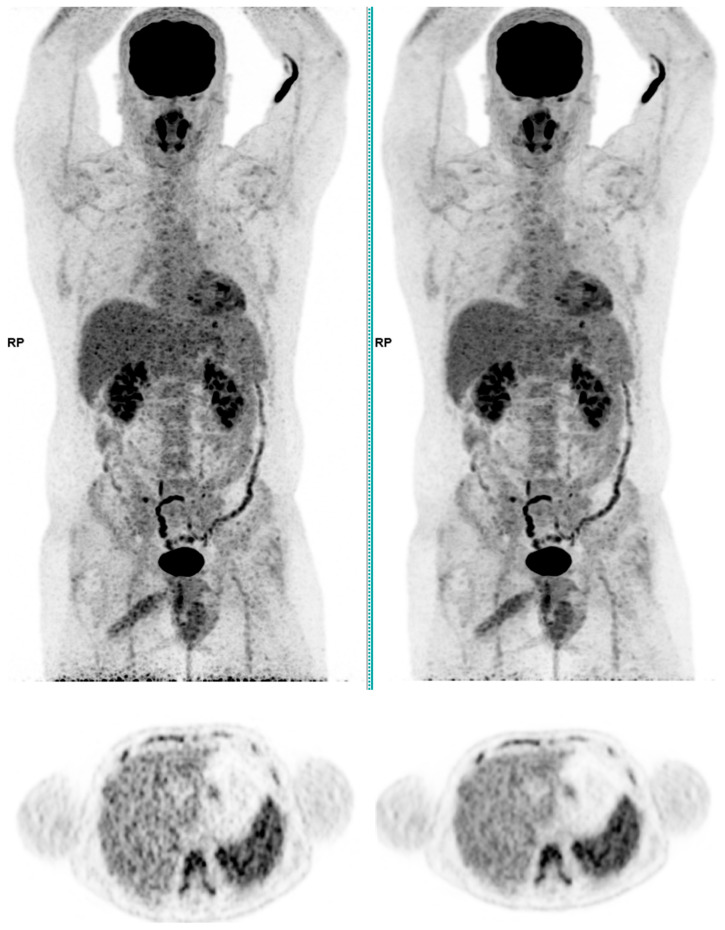
Total body 18F-FDG PET CT, MIP and transaxial images (**left**: without AI denoising; **right**: with AI denoising).

**Figure 3:**
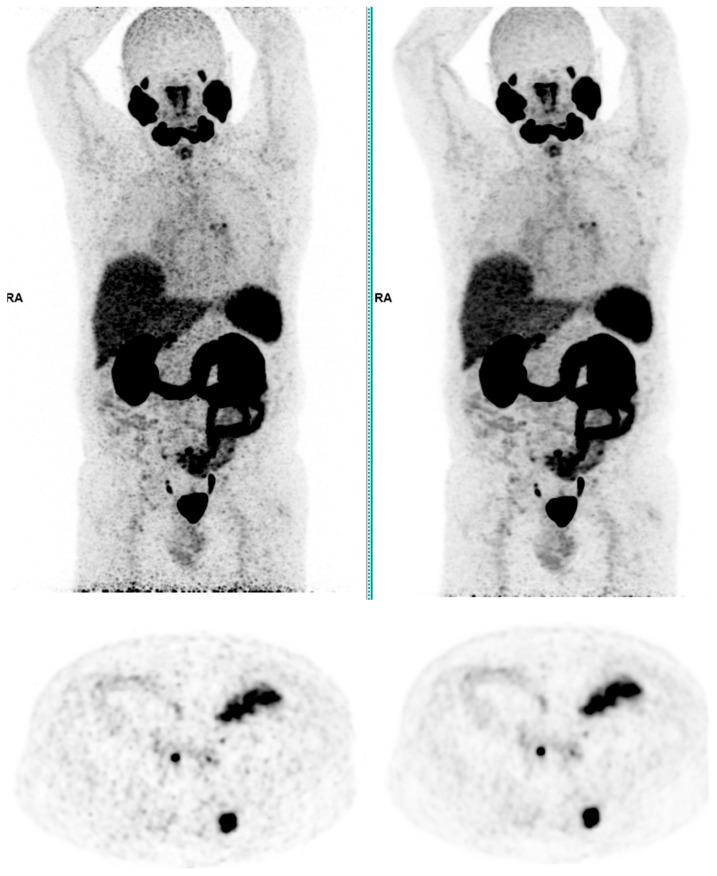
Total body 68Ga-PSMA-11 PET CT, MIP and transaxial images (**left**: without AI denoising; **right**: with AI denoising).

**Figure 4:**
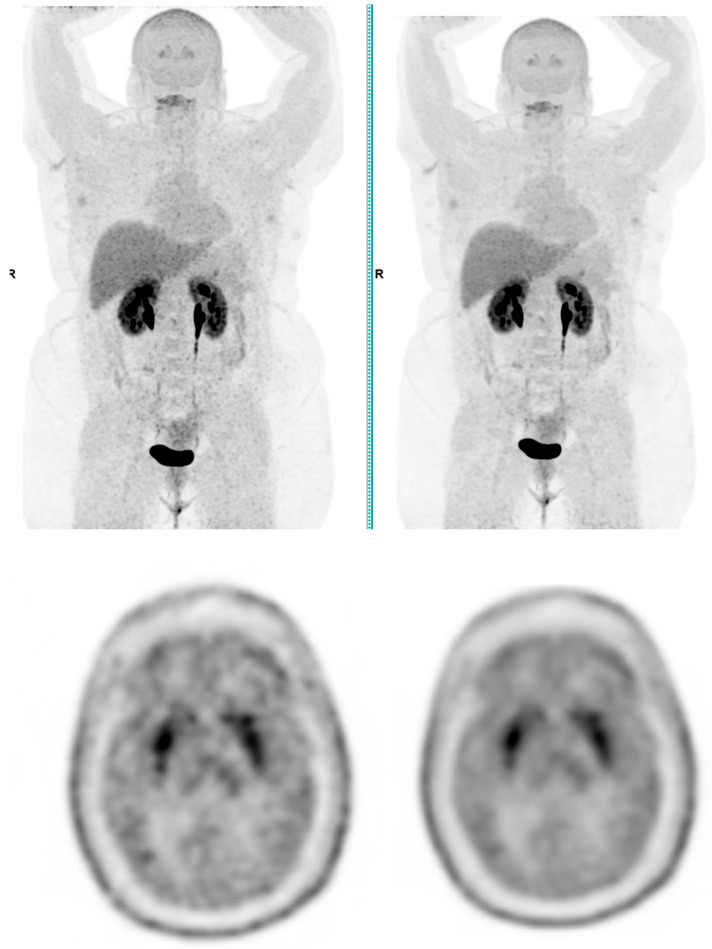
Total body 18F-FDOPA PET CT, MIP and transaxial images (**left**: without AI denoising; **right**: with AI denoising).

Across the three radiotracers, the AI-denoised images demonstrated a significantly improved performance in the visual analysis compared to conventional PET images. AI-denoised images were rated as interpretable (scores of 3–5) in all cases, compared to 65% for conventional images. Furthermore, an excellent image quality (score of 5) was achieved in 85% of all AI-denoised images, a significant improvement compared to the 50% in standard acquisitions. SubtlePET™ processing resulted in consistently lower noise levels, particularly for 18F-FDG scans, where 85% of images scored 4 or 5 for noise reduction compared to 60% in conventional scans. Lesion detectability was maintained or enhanced across all radiotracers, ensuring no compromise in the diagnostic accuracy. For 18F-FDOPA, a tracer with inherently lower signal-to-noise ratios, the AI algorithm improved the detection confidence by 25% compared to standard protocols. The visual comparison is illustrated through different MIP images and transaxial images (Figure 1, Figure 2, Figure 3 and Figure 4)

### 3.3. Quantitative Analysis of Image Quality

For ^18^F-FDG

**Figure 5:**
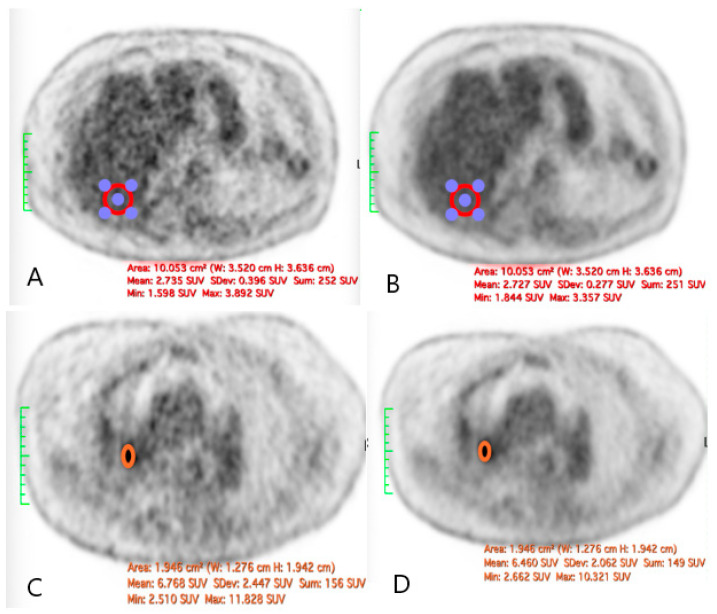
The illustration of a case of difference in the SNR before and after the application of SubtlePET™: ((**A**): liver VOI without AI denoising; (**B**): liver VOI with AI denoising; (**C**): right hilar lesion without AI denoising; (**D**): right hilar lesion with AI denoising).

**Figure 6:**
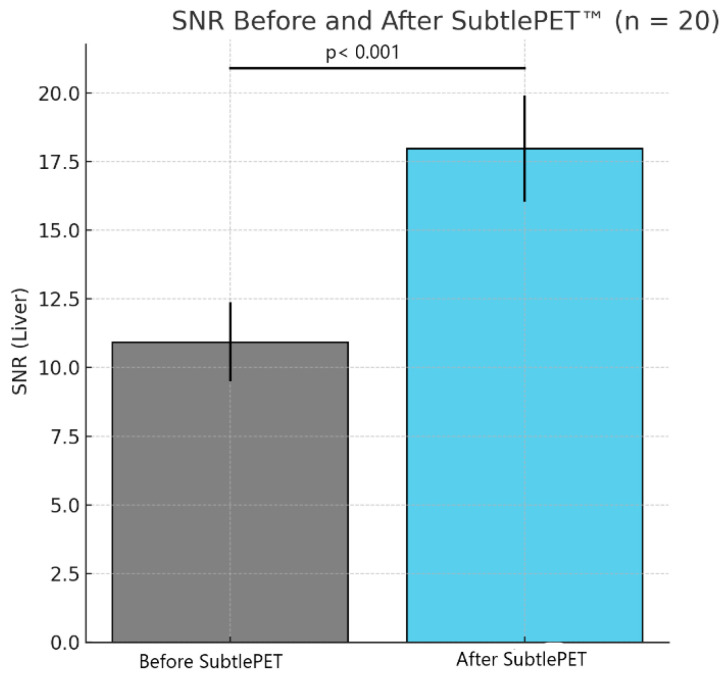
Comparison of SNR before and after SubtlePET™ application.

The quantitative assessment of the image quality demonstrated a significant enhancement following SubtlePET™ processing. The SNR improved from 11.2 in standard non-denoised images to 18.5 post-denoising. Similarly, the CNR increased from 5.1 to 8.9, reflecting an enhanced lesion conspicuity against the background activity. These objective metrics align with the improved Likert-scale image quality ratings and support the clinical utility of AI-based denoising in routine ^18^F-FDG PET imaging. An illustrative figure of a case of a right hilar lesion and a figure comparing the SNR before and after SubtlePET™ are provided (Figure 5 and Figure 6)

For ^68^Ga-PSMA-11

In the ^68^Ga-PSMA-11 subgroup, the application of SubtlePET™ also yielded notable quantitative improvements. The SNR rose from a baseline of 9.7 to 15.4 in AI-denoised images, while the CNR improved from 4.6 to 7.5. These gains were especially apparent in areas of moderate tracer uptake, such as pelvic lymph nodes, where subtle lesions became more readily discernible. This enhancement was consistent across BMI groups, indicating robust noise suppression and preserved diagnostic detail even at reduced acquisition times.

For ^18^F-FDOPA

For the ^18^F-FDOPA PET, which is inherently more susceptible to background noise due to a lower tracer uptake in certain brain regions, the SubtlePET™ processing significantly boosted the image quality. The SNR increased from 10.1 to 16.8, and the CNR improved from 4.9 to 8.1, facilitating improved visualization of the focal dopaminergic uptake in the basal ganglia and potential neoplastic lesions in the pancreas and adrenal glands. These enhancements are particularly valuable for interpreting subtle neuroendocrine abnormalities where a high image contrast is essential.

### 3.4. Reduction in Injected Activity

FDG (Table 2):

We have compared the FDG PET CT Total Body protocols in oncology indications for different BMI ranges (<25, 25–30, and >30), detailing the number of cases, mean injected activity (in MBq), and percentage decrease in injected activity. For BMI < 25, the “BODY FDG BMI-25/CHL” protocol (before) recorded 859 cases with a mean injected activity of 192 MBq, a 41% reduction in the injected activity compared to 1086 cases with 114 MBq in the “SUBTLE PET FDG BMI-25/CHL” protocol (after). For BMI 25–30, the “BODY FDG BMI 25–30/CHL” protocol (before) reported 511 cases with a mean injected activity of 245 MBq, a 32% reduction in the injected activity compared to 565 cases with 166 MBq in the “SUBTLE PET FDG BMI 25–30/CHL” protocol (after). For BMI > 30, the “BODY FDG BMI +30/CHL” protocol (before) recorded 379 cases with a mean injected activity of 340 MBq, a 16% reduction compared to 416 cases with 287 MBq in the “SUBTLE PET FDG BMI +30/CHL” protocol (after).

Ga-PSMA (Table 3)

For the PET CT Total Body Ga-PSMA, the “BODY 68GA-PSMA” protocol recorded 55 examinations with injected activity values of 86.58 MBq (min), 137.82 MBq (mean), and 173.53 MBq (max). The “SUBTLE PET 68GA-PSMA” protocol reported 57 examinations with reduced injected activity values of 60.68 MBq (min), 106.18 MBq (mean), and 144.67 MBq (max). The reduction factors in the injected activity between these two protocols were 0.70 (min), 0.77 (mean), and 0.83 (max).

### 3.5. Reduction in the Technologists’ Effective Doses (Table 4)

We have compared the third quartile daily PET dose in microSv for technologists (referred to as ATMs) between 2022 and 2023 across nine workers (ATM1 to ATM9). The horizontal axis represents the dose in microSv, and the vertical axis lists the workers. Blue bars indicate the third quartile dose for 2022, while orange bars represent the one for 2023.

Across most workers, the PET dose in 2023 is lower than in 2022, indicating an overall reduction in daily radiation exposure. Significant reductions are observed in ATM8, which shows a sharp decrease in the 2023 dose compared to its exceptionally high dose in 2022. Other workers, such as ATM6, ATM4, and ATM3, also demonstrate notable reductions in dose levels from 2022 to 2023. Workers like ATM9 exhibit minimal changes between the two years, indicating stable radiation exposure levels with only slight reductions.

Notably, ATM8 in 2022 stands out as an outlier, recording the highest dose among all workers and years, significantly exceeding not only his 2023 dose but also the doses recorded by other workers. The dramatic improvement for ATM8 suggests targeted interventions to address its previously high dose levels.

In summary, the overall reduction in the PET dose for 2023 reflects positive advancements in dose optimization and radiation safety protocols. These improvements likely result from enhanced procedural efficiencies or protective measures aimed at minimizing radiation exposure for workers. While the consistent performance observed for some workers, like ATM9, indicates stability, further efforts to ensure uniform dose reduction across all workers would enhance safety and compliance with radiation exposure guidelines.

**Table 4:** Comparison of technologists’ effective doses before and after SubtlePET™ implementation.

	Third Quartile 2022 (µSv) ± 95% CI	Third Quartile 2023 (µSv) ± 95% CI
ATM1	24 (21.6–26.4)	18 (16.4–19.6)
ATM2	24 (22.4–25.6)	18 (16.8–19.2)
ATM3	23 (21.4–24.6)	17 (15.6–18.4)
ATM4	24 (21.6–26.4)	15 (13.6–16.4)
ATM5	25 (22.6–27.4)	18 (15.5–20.5)
ATM6	23 (20.9–25.1)	20 (18.2–21.8)
ATM7	24 (21.5–26.5)	20 (18.0–22.0)
ATM8	52 (50.3–53.7)	31 (29.0–33.0)
ATM9	23 (20.9–25.1)	21 (19.9–22.1)

### 3.6. Impact on the Number of Patients (Table 5)

A comparative analysis was conducted to evaluate the variation in the number of patients before and after the implementation of SubtlePET, considering data from 77 days. The results indicate a notable increase in the patient volume following the adoption of SubtlePET. The results show a positive impact in terms of increased patient throughput. Before the implementation, the minimum number of patients recorded was 22, with a maximum of 29, a mean of 26.46, a median of 27, and a third quartile value of 28. After the adoption, the minimum number of patients increased to 24, with a maximum of 39, a mean of 31.41, a median of 32, and a third quartile of 33, indicating a significant improvement in the patient-handling capacity, with a mean increase of approximately 4.84 patients per day.

**Table 5:** Numbers of patients undergoing PET/CT per day.

Metric	Before SubtlePET	After SubtlePET
Number of patients per day: Minimum	22	24
Number of patients per day: Maximum	29	39
Number of patients per day: Median	27	32
Number of patients per day: Mean ± SD	26.46 ± 1.5	31.41 ± 2.0
Number of patients per day: 3rd quartile	28	33

## 4. Discussion

This study shows the good visual performance of the AI-denoised PET compared to the original PET in a digital PET/CT. Indeed, the AI-denoised images were universally interpretable (100% of cases with scores of 3–5), with a stark improvement from the 65% for conventional PET images. This indicates a significant leap in making clinical imaging more accessible and reliable for diagnostic purposes. Achieving “excellent image quality” (score of 5) in 85% of AI-denoised images compared to 50% in conventional acquisitions underscores the capacity of AI to enhance resolution and clarity. This quality boost likely stems from AI’s ability to suppress noise while retaining critical anatomical and functional details, which is essential in complex clinical cases. SubtlePET™’s ability to reduce noise is particularly noteworthy for its impact on 18F-FDG scans, where 85% of AI-denoised images scored 4 or 5 for noise reduction compared to 60% for standard scans. Noise reduction is pivotal for improving the visual assessment of PET images, especially in regions with low tracer uptake or for detecting small lesions. The maintenance or enhancement of lesion detectability across all radiotracers is a critical finding, as it reassures clinicians that AI processing does not compromise diagnostic accuracy. This is particularly relevant in oncological imaging, where accurate lesion identification directly impacts staging, treatment planning, and response evaluation. For 18F-FDOPA, which inherently suffers from lower signal-to-noise ratios, the AI algorithm improved the detection confidence by 25%. This is a remarkable achievement, as it addresses a specific limitation of this tracer and potentially expands its clinical utility. Improved detection confidence with 18F-FDOPA could enhance its application in neuro-oncology or movement disorder imaging, where precise delineation of pathological uptake is crucial. The study demonstrates that AI-denoised images not only improve technical parameters (e.g., noise reduction, visual quality) but also have tangible benefits in clinical outcomes, such as interpretability and lesion detectability. These results highlight the capability of SubtlePET™ in delivering superior visual quality and diagnostic reliability, supporting its integration into routine PET workflows to enhance both patient outcomes and operational efficiency.

Several studies have compared SubtlePET™ to traditional or advanced reconstruction-based denoising methods. Bonardel et al. demonstrated that SubtlePET™ outperformed standard low-count reconstructions and maintained an image quality comparable to a full-dose PET, with a CNR equivalent to full statistical acquisition using a Bayesian-penalized likelihood or OSEM with TOF [11]. Unlike reconstruction-based noise control, which often compromises quantification or requires complex implementation, SubtlePET™ allows for streamlined post-processing while preserving diagnostic confidence. Furthermore, it offers improved noise suppression compared to simple Gaussian filtering or wavelet-based post-processing methods, as shown in phantom and clinical studies.

Consistent with this, Quak et al. [13] demonstrated that the liver coefficient of variation (CV), an inverse proxy of SNR, decreased significantly from 15.57% in low-dose non-denoised images to 11.42% after SubtlePET™ processing (*p* < 0.0001). Similarly, the vascular CV dropped from 28.67% to 16.62% (*p* < 0.0001), representing a relative SNR gain of 27% to 42%. This objective improvement confirmed the visual Likert-based assessments and supports the robustness of SubtlePET’s denoising effect in reducing physiological noise while maintaining the diagnostic integrity.

Regarding the reduction in the injected activity, the “SUBTLE PET” protocol achieves a significant reduction in the injected activity for both the FDG and Ga-PSMA PET CT Total Body examinations while increasing the number of examinations performed daily. For the FDG, the mean reduction factor in the injected activity is 0.60, and for the Ga-PSMA, it is 0.77. This demonstrates an improved efficiency and reduced radiation exposure in the “SUBTLE PET” protocol for both imaging modalities.

As a direct consequence of the reduction in injected activity, across most workers, the PET dose in 2023 is lower than in 2022, signaling an overall reduction in the daily radiation exposure. This trend suggests that effective measures have been implemented to minimize technologists’ exposure to PET-related radiation. Significant reductions are evident in ATM8, whose 2023 dose shows a sharp decline compared to its exceptionally high dose in 2022. This marked improvement highlights efforts to address previously excessive exposure levels. Similarly, workers such as ATM6, ATM4, and ATM3 exhibit notable decreases in dose levels from 2022 to 2023, reflecting the broader success of interventions or procedural changes aimed at reducing the radiation exposure. Some workers, such as ATM9, exhibit minimal changes between the two years, indicating stable radiation exposure levels with only slight reductions. This stability might suggest consistent adherence to protective measures or the absence of significant changes in their work environment or practices. ATM8 in 2022 stands out as an outlier, recording the highest dose among all workers and years. This exceptionally high dose not only far exceeds ATM8’s 2023 levels but also surpasses the doses recorded by other workers. The dramatic improvement in ATM8’s exposure levels from 2022 to 2023 underscores the success of targeted interventions that address its previously elevated radiation levels, likely through tailored safety measures, revised protocols, or optimized workload distribution. The daily ATMs radiation doses are influenced by different tasks that were not included in this study (specific tasks, such as preparation of the radiopharmaceutical, injection, positioning and survey of the patient examination, and image acquisitions). Even more, this achievement has been obtained with a mean increase of approximately 4.84 patients per day.

Overall, the observed trends highlight the effectiveness of radiation safety strategies implemented between 2022 and 2023, contributing to enhanced occupational health for PET technologists. The data underscores the importance of continued monitoring and intervention to maintain and further reduce the radiation exposure in this high-risk occupational group.

Since its release in 2017, SubtlePET™ has transformed PET imaging by enabling up to 75%-faster scans using AI-powered denoising algorithms. This innovation reduces the imaging time while maintaining the diagnostic accuracy, offering improved support for diverse radiotracers in its latest version. Faster imaging enhances patient comfort by minimizing motionless periods, alleviating stress, and creating a better healthcare experience. It also has the potential to reduce motion artifacts thanks to shorter acquisition times. For medical facilities, SubtlePET™ boosts the efficiency by increasing patient throughput, optimizing resource use, and enhancing operational workflows. Successfully adopted by worldwide institutions, SubtlePET™ has improved the scanner performance, patient satisfaction, and clinical outcomes, cementing its role as a pivotal advancement in medical imaging.

The findings of this study align with and expand upon previous research into AI-based PET denoising, particularly regarding its clinical utility and operational benefits. SubtlePET™ consistently demonstrated its ability to enhance the image quality across all examined radiotracers. This observation is consistent with the findings of Quak et al., who reported significant noise reduction and improved tumor-to-background ratios in 68Ga-DOTATOC PET scans while maintaining the diagnostic accuracy [13]. Similarly, Weyts et al. showed that AI-denoised images for 18F-FDG PET achieved comparable or superior visual quality to standard images, even with a 50% reduction in the acquisition time. Our study further corroborates these results by demonstrating improved interpretability and diagnostic confidence, especially for tracers like 18F-FDOPA, which inherently have lower signal-to-noise ratios [10].

The reduction in the injected activity and acquisition time without compromising the image quality is a critical advancement. Our findings align with Bonardel et al., who validated SubtlePET™ for half-dose acquisitions, maintaining the lesion detectability and quantitative accuracy [11]. Furthermore, Margail et al. observed similar benefits in 68Ga PSMA-11 PET imaging, where AI denoising permitted dose reductions without a significant impact on the diagnostic performance. These parallels underscore the versatility and robustness of AI-driven denoising algorithms in optimizing clinical workflows [12].

Comparative studies further validate these findings, highlighting the distinct contributions of AI-driven software and advanced imaging hardware. For instance, the study led by López-Mora et al. demonstrated a 54% improvement in the image quality using digital PET systems compared to analog PET, with a marked advantage in detecting sub-centimeter lesions [14]. This complements another study by Katsari et al., which affirmed the non-inferiority of SubtlePET™ in producing diagnostically reliable images even at two-thirds of the standard dose for 18F-FDG PET scans [15]. Both studies illustrate how advancements in the imaging technology, whether through hardware or software, converge to achieve enhanced image quality and operational efficiency.

An important aspect of this study is the reduction in technologists’ radiation exposure, a factor not extensively addressed in prior literature. By decreasing injected activities and acquisition times, the study highlights operational safety benefits, complementing findings by Bonardel et al. on the reduction in radioactive exposure in low-count PET imaging. This aspect positions AI-based denoising as not only a tool for enhancing diagnostic imaging but also for improving occupational safety in nuclear medicine [11].

There are several limitations in this study. Firstly, the readers were not blinded, so they were aware of whether they were evaluating AI-generated or conventionally generated images, which could have introduced bias in the usage and evaluation of AI. We plan to include a blinded inter-reader study in future work. Secondly, one notable limitation of AI-based denoising techniques, such as those applied in this study, is the potential for over-smoothing, which may lead to the blurring or even loss of subtle lesions. This phenomenon, sometimes referred to as “AI hallucination,” raises concerns regarding the reliability of lesion detectability, especially in diagnostically critical regions. While our visual assessments did not reveal any false negatives attributable to such effects, the risk remains an important consideration. Therefore, we have expanded our discussion to acknowledge this limitation. Future large-scale, multi-reader validation studies will be essential to rigorously assess whether these smoothing artifacts may compromise the diagnostic accuracy, particularly in low-contrast or small lesions.

## 5. Conclusions

In conclusion, this study highlights the transformative impact of SubtlePET™ on PET imaging, showcasing its ability to enhance image quality, optimize operational workflows, and improve occupational safety. By leveraging advanced AI-based denoising algorithms, SubtlePET™ effectively reduces acquisition times and injected radiotracer activities without compromising the diagnostic accuracy. These advancements have been validated across multiple radiotracers, including 18F-FDG, 68Ga PSMA-11, and 18F-FDOPA, demonstrating consistent improvements in image interpretability and lesion detectability.

Furthermore, the significant reduction in the technologists’ radiation exposure underscores the dual benefit of improving both patient and staff safety. The findings align with existing research while expanding on the clinical and operational advantages of AI-driven PET-denoising technologies. As the demand for PET imaging continues to grow, integrating tools like SubtlePET™ represents a critical step toward achieving higher patient throughput, enhanced diagnostic confidence, and streamlined nuclear medicine practices.

In total, this study underscores the importance of adopting AI innovations to meet the evolving needs of healthcare systems while maintaining the highest standards of imaging quality and safety. Future research should focus on broader multicentric validations, exploring the application of AI-based denoising in diverse clinical settings and with emerging radiotracers.

## Figures and Tables

**Table 1:** Main patient characteristics.

Radiotracer	Number of Cases	Mean Age ± Standard Deviation (Years)	Main Clinical Indications
FDG	3816	61.2 ± 11.0	General oncology: lung, breast, lymphoma, colorectal, ENT
PSMA	112	71.5 ± 7.5	Prostate cancer
FDOPA	18	58.0 ± 13.0	Neuro-oncology and neuroendocrine tumors

FDG: fluorodeoxyglucose; PSMA: prostate-specific membrane antigen; FDOPA: fluorodopa.

**Table 2:** FDG PET/CT Total Body: injected activity by BMI group changes.

Protocol	BMI Group	Timing	Number of Cases	Mean Activity Injected ± DS (MBq) and IC95	% Decrease
FDG	<25	Before	859	192 ± 24.0 (190.4–193.6)	
SubtlePET FDG		After	1086	114 ± 14.3 (113.1–114.9)	41%
FDG	25–30	Before	511	245 ± 30.6 (242.3–247.7)	
SubtlePET FDG		After	565	166 ± 20.8 (164.3–167.7)	32%
FDG	>30	Before	379	340 ± 42.5 (335.7–344.3)	
SubtlePET FDG		After	416	287 ± 35.9 (283.5–290.5)	16%

**Table 3:** Ga-PSMA PET/CT Total Body: injected activity changes.

Protocol	Timing	Number of Cases	Mean Activity Injected (MBq)	% Decrease
BODY 68Ga-PSMA	Before	55	137.82 ± 17.23	
SUBTLE PET 68Ga-PSMA	After	57	106.18 ± 13.27	25%

## Data Availability

The datasets presented in this article are not readily available due to ethical restrictions.
